# Atlanta Academy of Medicine

**Published:** 1877-06

**Authors:** 


### Reports of Societies.

### ATLANTA ACADEMY OF MEDICINE.

ATLANTA, GA., May, 8, 1877.

Vice-President A. W. Calhoun in the chair.

Dr. R. W. Westmoreland presented, for the inspection of the Academy, a fatty tumor which had been removed from the upper portion of the thigh. The pathological specimen was presented for the purpose of calling their attention to Lister's antiseptic method, which had been used during the operation. Carbolic spray (composed of one part of carbolic acid to twenty of water) was thrown constantly upon the part, from the commencement of the operation to its completion, and the instruments and hands of the operator washed in it. The incision for removal of the tumor was eight inches long. The two edges of the wound were brought together by sutures then covered with a cloth saturated with a solution of carbolic acid. Three days has elapsed since the operation, and there is no evidence of untoward inflammation. The parts have the appearance of uniting by the first intention.

Dr. W. F. Westmoreland: There is no doubt that the antiseptic treatment, as practiced by Lister, will prevent suppurative inflammation, under favorable circumstances. The reports of European surgeons are very favorable. In large lacerated wounds, they have found the carbolic dressing especially beneficial.

Dr. J. T. Johnson thought that it would require a wound of more serious character to constitute a test of the value of the method.

Dr. J. G. Westmoreland said that the object of applying the antiseptic by European surgeons, seemed to be that of destroying bacteria, which they suppose, by coming in contact with the injured part, lead to inflammation. He believes carbolic acid will prevent putrefaction and perhaps inflammation, but thinks, as the former often occurs in deep-seated parts that are protected from contact with the supposed organism, that it is not due to bacteria. In fact, putrefaction in dead animals generally commences at the bone. It is a natural process, after the loss of vitality, but may be arrested, or prevented, by the presence of carbolic acid.

Dr. W. F. W. said: He thought that Dr. John W. did not understand what Dr. Lister claimed for this method. Pus may occur from a high grade of inflammation. He remarked that, if we take milk, and excluded these molecules, or organisms, we prevent putrefaction. It is not the atmosphere that produces degeneration, but the organisms that float freely in it, and coming in contact with certain substances produce decay.

Dr. V. H. Taliaferro called the attention of the Academy to the antiseptic properties of turpentine. He thinks that it is equally as effective as carbolic acid, when applied to wounds, and has the advantage of always being readily obtained, and is much cheaper.

Dr. W. F. Westmoreland reported a very unique and interesting case. He was called to Fayetteville, about a week ago, to see a stout, robust man of middle life. Upon examination, the scrotum and penis were found to be of immense size from the effusion of serum. The distension was the result of an accute inflammation. He had been a sufferer for the last 18 months. He had urinated more or less freely up to the outburst of the acute inflammation, and since that time he has had obstruction of urine. The treatment was commenced with the idea that there was an infiltration of urine into the scrotum and cellular tissue. Numerous small incision were made in the scrotum, followed by a profuse flow of urine and pus, but without pain. His condition was alarming, the constitutional signs indicated the gravity of his disease. Gangrenous inflammation had also begun as was shown by the blackening and insensibility of the scrotum. An endeavor was made to save as much tissue as possible, by freely puncturing and incising the greatly distended tissues. Large portions of the scrotum sloughed off, notwithstanding the punctures and incisions. The condition at present is very favorable. He has passed through the trying ordeal with the following lesions: the loss of his scrotum and of the bulbous portion of the urethra, or more properly the tissue surrounding the bulb. The catheter can now be passed with perfect facility, and the urine drawn off. The diet throughout has been very nutritious.

Dr. W. F. W. spoke of the nitrate of lead as an antiseptic. He had found it effective where other antiseptics had failed.

Dr. J. M. Johnson: Was called, on the 23d February, at four o'clock A.M, to see Mrs. L., of West End, at confinement with her third child. He found a large bag of water, which he ruptured, and the labor terminated in a few minutes. As soon as the child was disposed of, he turned his attention to delivering the after-birth, and found that the womb, which had contracted well at first, had receded, and could not be felt except above the navel. By external manipulations he succeeded in inducing pretty firm contraction, and upon examination found the placenta decending, but attended with considerable hemorrhage, which he thought was the blood contained in the placenta. Gentle traction was made on the cord, and finally the forefinger was hitched into a loop of the placenta, which gave way at a very gentle pull, and the blood flowed so furiously that he introduced his left hand to the fundus, which had receded to the position first described, and with his right, grasped it, and seizing the afterbirth withdrew it. The contraction lasted but for a moment however, when the womb receded again and the blood flowed as before. He then discovered that the placenta was delivered entire, but the membranes remained. He again thrust his hand to the fundus, to find the membranes so firmly adherent that they had to be scraped off with the finger nail. He began this operation, working as vigorously as possible, when the womb suddenly contracted again and expelled his hand, bringing away less than half of the adhearing membranes, and attended with an immense discharge of blood. Fifteen minutes had now alapsed, and his patient had lost half the blood in her body. Ice, which had to be sent for, a distance of four hundred yards, arrived at this stage of the trouble, and a piece nearly as large as a goose egg, was introduced to the seat of difficulty and passed rapidly round the entire surface of the womb; contraction again followed, but did not detach the membranes. In two or three minutes the womb receded again, and he again introduced his hand with a large lump of ice, which he turned loose and resumed work on the adhearing membranes with his finger nail. At the next contraction, four-fifths had been removed, and at the next relaxation, the last vestige was removed, and the entire surface well explored to make sure that nothing was retained. Here he expected his trouble to end, but it did not. The womb continued to relax and contract in this manner for two hours and a half, and he kept his hand, with a lump of ice as large as a goose egg, continually in the womb, all that time, except when expelled. He passed his hand with the ice into the womb to the fundus more than twenty times before the womb became permanently contrated. In conclusion, he said that nearly or quite ten pounds of ice was consumed, the patient became pulseless, and nearly every drop of blood had apparently drained away, but she rallied rapidly, and has had as good a recovery as he could ask.

Dr. Johnson is of opinion that many lives might be saved by the boldness here practised. It would seem to be not generally known that the womb is large enough for the hand of the accoucheur, or at least he rarely hears or reads of such an exploit. In his opinion the hand may be harmlessly and beneficially introduced into the womb, even to remove clots, to say nothing of the adhering secondines. In a practice of forty-six years, he has not met with a case like it.

Dr. Taliaferro said he was at a loss to determine in what Dr. Johnson's case was peculiar. Was it in the inertia? That was common. Was it in the hemorrhage? that was not unfrequent. Was it the retention of a portion of the membranes, chorion and amnion, that occured so frequently, and made so little difference whether it came off or not, that he hardly considered it a factor in the case. The lochia will wash these away, for some portions he thought was more than apt to be left behind; of course he did not allude to the placenta as a membrane or part of them.

Dr. W. F. Westmoreland said that he thought it was a matter of no little moment. For a piece, the size of a pea was sufficient to cause septicæmia, and a clot of blood smaller in bulk than the membranes, had given rise to, and been the cause of continuous recurring and even fatal hemorrhage.

[NOTE.—The above report and comments thereon present, indeed, an anomalous affair. We can conceive of a portion of the placenta remaining adherent to the uterus, but cannot understand how the membranes can *adhere* after the placenta has been removed entire.—EDS.]

---

J. S. TODD, M.D., REPORTER.

---

ATLANTA, May 15, 1877.

V. H. Taliaferro, M.D., President, presiding.

Upon discussing the entertaining essay, printed elsewhere, by Dr. Henry L. Wilson, on mania a potu,

Dr. Jno. G. Westmorland favors the idea that this condition is brought about by the withdrawal of the stimulant.

Dr. Baird was distinctly of the opinion that delirium tremens was caused by both the withdrawal and continuance of the liquor; i. e. a long continued excessive use of the alcoholic liquors would give rise to this condition; so would the sudden cessation.

These opposite conditions, causing the same disease, has in his opinion been the cause of the diversity of opinions as to the treatment with or without stimulants, alcoholic in character. He had practiced and would advise the giving of whisky to a case where its withdrawal had been the exciting cause of the attack, on the other hand, where the delirum tremens was consequent upon a debauch, he would prohibit its use. Where it was necessary to administer alcoholic stimulants, he would gradually lessen the quantity and endeavor to procure sleep by the administration of opium. In these forms of the affection the result of excessive ingestion, of alcohol, he placed confidence in large doses of the bromide of potass. one drachm, hydrate of chloral 10 to 20 grains, repeated as required.

Dr. Westmoreland asked Dr. Baird if his remarks were the convictions of his observations of the disease, or the result of reading.

Dr. Baird: Both observation and research taught him that he was correct.

Dr. Westmoreland could not understand how the absence of alcohol should produce the same train of symptoms as its excessive use. He always withholds it in treating the disease. He believes that the disease would sometimes be arrested by the giving of alcohol alone, but it would lead to it in the end, therefore he does not give it at all. Until sleep is secured no medication will be of any avail towards a cure. He stimulates the brain with opium, chloroform, chloral and the like.

Dr. J. M. Johnson's observation, which has been extensive, coincides with those of Dr. Baird, and accordingly he sometimes gives and sometimes withholds whisky. He gives a large purgative dose of calomel, also opium, bromides, chloral, and when the pulse is rapid and strong tinct. varatum viride; has also found alkalies, such as magnesia and chalk of service in allaying the irritable condition of the stomach. Just cured a case where the mania came on six or eight days after all potations had ceased, and which required ten or twelve doses of morphia, a grain each to induce sleep.

Dr. W. F. Westmoreland said he thought there were two varieties of the affection, but his views were not well defined. He believes that the withdrawal is more frequently the cause, than excessive ingestion. A man may continue to drink, drink excessively, but his digestive organs be in such a disordered state that the stimulous will not be appropriated. This is the same as withholding it, for it does not go into the blood. To control the circulation is a desideratum devoutly to be hoped for. For this purpose in England digitalis in half ounce doses of the tincture have been repeatedly given, and the reports of its good effects are almost universal. Dr. Dugas, of Augusta, uses the cold douche to accomplish the same end. With him varatrum takes precedence. He has found that all agents act better after mercurial. Considers it the shortest way to secure sleep and cure the case, to give calomel and then the anodynes.

Dr. Johnson said to Dr. Wesmoreland, I would not let it go out to the country with my apparent sanction, for he was satisfied Dr. Westmoreland did not mean it, that he would advise the use of tinct. digitalis in half oz. doses. He was satisfied that it would be the cause of some funerals. He would relieve, and endeavor to put to sleep with 20 to 30 gr. doses of chloral and drachm doses of bromide potass., and not wait for the purgation. Let it come on afterwards. Advised members not to carry the cold douche to the back of the head too far, less you bring in a colapse which will give you trouble.

Dr. Westmoreland: The dose of digitalis he had mentioneed was a standard remedy in England. He had never given it himself however.

Dr. Johnson was glad to hear him say so.

Dr. Baird alluded to a case where a patient had all the symptoms of mania a potu from excessive drinking during the day, absinthe was also in the last potation. Has he to wait in this case for the action of a purgative? He thought not, and gave 30 or 40 drops of tinct. opii, 30 grs. potass. bro. and 20 chloral hydrate, and in a very short while he was asleep and required no further attention.

Dr. Westmoreland considered his patient drunk; nothing more than wild from excessive stimulation, and not mania a potu.

Dr. Baird said he thought it a typical case of mania.

Dr. Jno. G. Westmoreland: Why did you not give whisky?

Dr. Baird: Because it was of the very class of cases which being produced by too much alcohol, was to be treated by not giving it.

Dr. Todd thought Dr. Baird's etiology of the disease correct, and endorses his treatment as to the alcohol, etc. He thought that the administration of calomel was essential when you had a well developed case, which was nearly always attended by constipation, dry tongue, etc. The failure of large doses of opium, chloral, etc., to produce sleep was because they were not absorbed. You must unlock the portal circulation before digestion can take place. As vomiting is generally a prominent symptom, and veratrum an emetic, if he gave it at all, should do so hypodermically. Calomel not only acts on the liver, but allays in large or very small doses, irritability of the stomach.

Dr. McIntosh asked Dr. Todd if he thought calomel would be any more likely to be absorbed, it being insoluble, than other medicines, such as morphia, bromides and chloral, which were so soluble? and also asked if calomel could act before it was taken up, i. e. absorbed.

Dr. Todd said there was something inexplicable about the good results obtained from calomel. He did not believe that any medicine could benefit a constitutional malady unless it was first absorbed. The older authors claimed that mercurials acted upon the liver by stimulating the duodenal orifice of the ductus communis choledocus. If this is true, bile being the natural peristaltic stimulus to the intestines, and playing an important part in digestion, then it was possible for calomel to act before being absorbed. He was glad Dr. McIntosh asked him the question, and believed it to be a fundamental truth that medicines must be absorbed to be of benefit to other than local affections.

Dr. J. G. Westmoreland: Although cantharides may be absorbed from the skin and affect other parts after entering the circulation, its blistering effect is made by local action, and may be had without absorption. Do you believe that it is absorbed in order to form a blister?

Dr. Todd: *Most assuredly.* No blister is formed until absorption of some of the flies takes place. Strangary often follows the application of flies, which could not occur unless it had found its way into the blood. The difficulty of obtaining a blister during high fever, or collapse is also confirmative of his views. The perverted state of the absorbents of the skin prevented its absorption during these conditions, and the result was no blister, until the fever abated, or reaction was established.

Dr. Todd thought calomel less likely to be taken up than morphia, bromides or chloral, in fact was absorbed with more difficulty than any other remedy.

Dr. Jno. M. Johnson: Calomel will act by its very weight. Its absorption was not necessary as a purgative.

Dr. Todd: The weight will not carry it up the ascending colon.

Dr. Johnson: Had known of a man to swallow a set of teeth and they got up it, and were voided per annum. Fluids are very apt to be absorbed without any reference to the condition of the bowels. He knew of a woman, whose stomach digested enough nutriment to keep her alive for several years, and her bowels only acted twice in a year. Saccharated calomel acts in small quantities because it goes directly into the circulation. The attrition with the sugar reduces the particles so small that they find entrance into the mouths of the absorbents.

---

ATLANTA, May 22, 1877.

Vice President, Dr. A. W. Calhoun, in the chair.

Dr. J. T. Johnson read an essay on small congenital urethal meatus.

Dr. Simpson had recently given prompt and permanent relief to a patient who has been treated for piles. He found upon examination that he was the subject of an anal fissure, which he forcibly dilated. He thought this operation preferable to that of dividing the sphincter with a knife, because occasionally the latter operation was followed by inability to retain the fæces, and gases of the intestines on account of the failure of union of the divided muscular fibres.

Dr. Todd said he considered it a question of some moment to determine which was the better operation, forcible dilatation or cutting, and asked an expression of the members.

Dr. J. T. Johnson said that permanent paralysis of the sphincter following either operation was so rare that he had not been able from reading or observation, to decide which was the more likely to give troublesome sequences.

Dr. J. G. Westmoreland: Reasoning from analogy, was inclined to think that as a clean cut healed more readily than a contused or torne wound, that the cutting would be therefore less apt to give paralysis of a permanent character, and less likely to cure the fissure.

Academy adjourned.

O. R. FORD, M.D. REPORTER.

---

ATLANTA, May 29, 1877.

Dr. A. W. Calhoun, second Vice-President, presiding.

Dr. W. F. Westmoreland reported a case of interest and importance. The subject, a well known physician of this State, was bitten by an angry bitch, about nine weeks ago.

The animal had been spayed by the Doctor, and died in a day or two after inflicting the wound, of peritonitis, the result of the operation.

The bite amounted simply to an abrasion on the forefinger of the right hand. No importance was attached to it, and it healed kindly. Shortly after this, the other hand was slightly wounded by a spicula of bone, in an operation upon a horse, followed in a few days by neuralgic pains, radiating from the point of injury. These, however, subsided spontaneously in less than a week. About three weeks after the reception of the bite, irregular, shooting pains commenced in the finger, hand, arm and shoulder, of the right side. These pains soon became very intense, and while they have not been continuous, there have been, constantly, various uneasy sensations, as heaviness, burning, twitching, etc., in the affected limb. The usual remedies for neuralgia have been used in rotation without appreciable benefit. Iron, quinine, strychnia, phosphorus, etc., have been faithfully tried. Opium, which it has been necessary to use on account of the severity of the pain, secures only a temporary freedom from suffering. He is now using the constant galvanic current to the arm, and has obtained more relief from it than from any other remedy.

What is the cause of the difficulty?

He expressed the belief that the train of symptoms observed were due to inoculation with the saliva of an angry dog. He referred casually to the case of a gentleman who was bitten several years ago by a rat. He suffered severely from the most alarming train of nervous symptoms, for six or eight weeks, but finally recovered. He presented the two cases as in many points analogous, and said he believed the symptoms were in both cases the result of inoculation with poisonous saliva, and that the saliva was poisonous because the animals were angry.

It is claimed that hydrophobia may result from the bite of an angry dog, or a bitch in heat, and that it is not necessary that the animal should be rabid, for the production of the disease. He is of the opinion that this is true. He believes that the quality of the saliva is changed by passion, and that it is increased in quantity. A bite from a dog, under these circumstances, may be followed by a train of nervous symptoms identical with, or closely simulating hydrophobia. This is said to be proven as to the Spitz dog, and statistics show an increase of fifty per cent. in cases of hydrophobia, since their introduction into this country.

Dr. John G. Westmoreland said the temper of the animal, and the quality of the saliva, had nothing to do with the symptoms, in the case of the gentleman bitten by the rat. He saw the case alluded to—had attributed the symptoms observed to septicæmia. He had known similar consequence ensue from a puncture by a pin. The peculiar character of the wounds, being narrow and deep, favors this result. Thinks that hydrophobia and tetanus are often counfounded. Once saw a man die of tetanus, after being bitten by a dog.

Dr. W. F. Westmoreland remarked that the history of the case was not one of septic poisoning, purely. Trivial wounds are frequently sustained without any following disturbance, but the bite of a rabid animal results in the most distressing symptoms. It is the poison that is introduced, and not the wound, that does the damage.

Dr. Baird was familiar with the case reported by Dr. Westmoreland, and disagreed with him, both as to the cause and nature of the affection. Unfortunately, for the sake of his argument, it is based solely upon hypotheses. He offers no evidence in support of his belief that the symptoms detailed are the result of the bite of the bitch—the opinion advanced that emotional activity renders the saliva poisonous is by no means established, and the theory that hydrophobia, or even abnormal nervous phenomena, may result from the bite of an animal not rabid himself is, to say the least of it, open to grave doubts. The gentleman certainly has not the symptoms of hydrophobia, so the considervtion of that disease may be, at once, discarded. If the disturbance observed was the result of a poison taken into the circulation, as alleged by Dr. Westmoreland, it seems very remarkable that, acting as it must, through the nervous centers, that symptoms should be limited to deragements of sensibility in one limb. It is surely not a common state of things to find the blood charged with "poison" and the individual in robust health, with all the functions of the body normally performed, and the only evidence of the existence of a serious constitutional infection, a neuralgia of some of the nerves of one arm! He could understand how a wound of a nerve trunk, or even a nevous filament, might result in an inflammation of the sheath, and a protracted source of irritation thus becomes established, but in this particular case, the hyperæsthesia is not confined to a single nerve, and is not fixed in any number of nerves, but shifts its position irregularly, from place to place, and this fact, together with the intensity of the pain experienced, convinces him that the mind is an important element in the causation of the trouble. He believes there is some neuralgia, but that it has not been caused by the bite; this fact however, has induced the patient to exaggerate sensations observed, that, under ordinary circumstances, would not have attracted attention. He thinks the disease is neuralgia, and pathaphobia. He gave a very favorable prognosis.

Dr. W. F. Westmorland added that there had been some fever and derangements of the digestion.

Dr. Todd observed that he had never known it claimed that one angry dog could produce hydrophobia in another dog. It is impossible to imagine a higher degree of passion than is exhibited by two dogs fighting each other; yet hydrophobia has never been developed from this source. Why should it follow the bite of an angry dog in the human subject? He cannot subscribe to the doctrine. Hydrophobia seems to be the fashion in New York, and statistics feels its influence.

Dr. Baird added that Spitz dogs are the fashion, and if women will have dogs instead of babies, they must expect to suffer the real or imaginary penalties that attach to the unnatural fancy.

Dr. J. G. Westmoreland desired to call the attention of members to the following case of meningitis: On the 12th of May, a sprightly, intellectual girl, some eight or ten years old, complained of feeling unwell in the afternoon, and at night grew worse, with perverted vision and other unpleasant symptoms. Objects were seen double and inverted. Fever, restlessness, opisthotonos, delirium, and a sense of suffocation, or *choking*, as the patient expressed it, ensued. Was called at 2½ o'clock, two or three days after these symptoms were first manifested, and found the little girl flouncing and writhing, with various contortions of the body and limbs, with occasional complaint of choking, which was about the only word spoken distinctly. The pupils were widely dilated, the skin hot, and the pulse moderately full and frequent. No opisthotonos was present at the time, but, as reported, had been a prominent symptom the previous night. Symptoms of suffocation soon became alarming, and in thirty minutes after seeing the patient, breathing ceased entirely, the face and lips exhibiting the purple hue of asphyxia. Artificial respiration was kept up two or three minutes, but ascertaining that the regular process was not established, it was continued, in all, at least five minutes, before artificial means could be dispensed with. Evidently asphyxia was from paralysis of the pneumogastric nerve.

The treatment consisted of a blister to back of head and neck, which was applied the previous night, the abstraction of blood from the arm, thirty grains of calomel, and the eighth of a grain of morphine hypodermically. This was commenced immediately on my arrival.

The patient was comfortable in two hours, ond rested well during the night, fever gradually abating, so that very little was present next morning.

Some rise of fever during the afternoon of the second day, and tendency to restlessness and muscular agitation during the night concluded the unpleasant symptoms, and the patient rapidly recovered.

He asked members to name the disease, and had in reply the opinion of its being an unmistakable case of cerebro-spinal meningitis, from which no one dissented.

